# Inland Waterways and Population Health and Wellbeing: A Cross-Sectional Study of Waterway Users in the UK

**DOI:** 10.3390/ijerph192113809

**Published:** 2022-10-24

**Authors:** Nafsika Afentou, Patrick Moore, Katrina Hull, Jenny Shepherd, Stephanie Elliott, Emma Frew

**Affiliations:** 1 Health Economics Unit, University of Birmingham, Birmingham B15 2TT, UK; 2 Health Economics, University of Bristol, Bristol BS8 1QU, UK; 3 Canal & River Trust, Milton Keynes MK9 1BB, UK

**Keywords:** inland waterways, blue space, physical activity, mental wellbeing, life satisfaction, travel cost method

## Abstract

Natural environments, such as inland waterways (IWs), have been identified as a potential means to increase physical activity and promote health and wellbeing. However, further information on predictors of IW usage and their relationship with health and wellbeing outcomes is needed. Data were taken from the cross-sectional UK Waterways Engagement Monitor survey of waterway users (n = 21,537) in 2019/2020. Health outcome measures were life satisfaction, physical activity, and mental wellbeing. Visit frequency was an additional outcome measure. Both bivariate and multivariable associations between outcome measures and features of IWs were explored. The travel-cost method was used to estimate users’ demand, expressed by travel costs to waterways. Multivariable models showed positive associations of frequent visits and use for recreational/leisure purposes with life satisfaction and physical activity. Rural visits were associated with higher life satisfaction than urban ones. Lower visit satisfaction negatively impacted life satisfaction and mental wellbeing. Visit frequency was influenced by individual characteristics and purpose of visit, including visits for exercise. Waterway visits were inversely associated with travel costs (IRR = 0.99, *p*-value ≤ 0.001), and there was greater demand elasticity for short distances (≤5 miles). Socioeconomic-related inequalities were present. Future policies could enhance frequent use of waterways and alleviate accessibility-related inequalities to improve population health outcomes.

## 1. Introduction

In recent years, there has been a growing body of literature identifying natural environments (e.g., parks, woodlands, waterways) as a means to promote health and wellbeing in the population [1,2,3]. Three main mechanisms have been proposed which link the presence of natural environments to health-related benefits: benefits through physical activity (PA), benefits through social interaction, and psychological benefits such as relaxation, mental restoration or relief from fatigue [4]. According to existing evidence, proximity and exposure to green spaces have been independently associated with improved self-reported wellbeing [5,6], lower levels of stress [7], reduced symptoms of depression and anxiety [8,9], and increased levels of PA [10,11,12]. In several studies, outdoor PA (such as walking, running or cycling) led to better mental health and wellbeing outcomes compared to indoor PA, thus mediating the association between natural environments and health [1,4,13]. Compared to exercising indoors, PA in natural settings was associated with greater engagement [14] and psychological benefits from the restorative properties of nature [15].

Whilst research has concentrated on the health impact of green space, fewer studies have focused on blue space as a protective health factor in local natural settings. Blue space refers to natural or manmade outdoor water surfaces (e.g., lakes, rivers, canals) that are visible or proximally accessible to humans [16]. Previous reviews suggest potential health benefits of outdoor blue spaces in relation to physical activity, life satisfaction, and mental wellbeing, yet the results are inconsistent [17,18]. The robustness of findings is mainly challenged by the limited number of available studies and the heterogeneity of methodological approaches. In England, the availability of inland waterways (IWs) has been linked to better mental health with no mediating effect by PA [19]. Lack of access and availability of IWs seems to burden populations with low socioeconomic status who live in deprived areas, thus escalating health inequalities [20].

In the absence of user fees, user demand is reflected in the association between number of visits and travel costs, which is used to derive the average willingness to pay for visiting the IWs. Investment in the maintenance and accessibility of IWs can increase visits and user numbers, which in turn may lead to improvement in health and wellbeing [21]. Given existing budget constraints for the restoration and upkeep of IWs, a better understanding of the links between IW usage, user demand, and subsequent benefits to population physical and psychological health is needed to inform investment decisions.

Therefore, the aims of this study were (i) to explore the relationship between the features of IWs and population physical and mental health and wellbeing, (ii) to examine predictors of visit frequency, and (iii) to generate evidence on the average willingness to pay for visiting the IWs as reflected by user demand.

## 2. Materials and Methods

Statistical analyses were conducted to estimate the association between population health and common ‘features’ of IWs and to identify predictors of visit frequency. A travel cost method [22] was used to examine user demand.

### 2.1. Data Collection

The data were from the Waterways Engagement Monitor (WEM), a survey conducted by the Canal & River Trust (Trust), which is responsible for maintaining the inland waterway network in England and Wales, including surrounding infrastructure (e.g., towpaths). The WEM collects monthly cross-sectional data on adult (>16 years) waterway visitors to assess outcomes on wellbeing, physical and mental health, and motivations and barriers to use. The sampling strategy uses an online panel methodology for recruiting individuals with common characteristics on-site through a ‘by-invitation-only’ proprietary method. Participants are asked about their lifestyle, physical activity, mental wellbeing, life satisfaction, and use of IWs. Other data included socioeconomic status and demographic characteristics. Data used here were collected between January 2019 and April 2020.

### 2.2. Outcome Measures

Physical health and wellbeing were assessed using three different outcome measures: life satisfaction, physical activity, and mental wellbeing.

Life satisfaction (LS) was measured using a 10-item scale [23] with zero corresponding to the lowest level and 10 to the highest possible level. Mental wellbeing was estimated using the validated Short Warwick–Edinburgh Mental Wellbeing Scale (SWEMWBS) © [24]. Original scores were transformed to a metric scale and ranged from 7 (lowest wellbeing) to 35 (highest wellbeing).

In the WEM survey, participants were asked to report the type of activity they undertook during their visits to waterways from an answer list. The list comprised boat trips, fishing, exercise (running, walking with/without a dog, cycling, water sports), commuting elsewhere, heritage visits, buying food/drink, sitting/standing by water for relaxation, and other visits. PA data were collected by asking the number of ‘days over the past month’ that participants walked, cycled, or participated in water-based sports or other exercise. National guidelines recommend at least 150 min of moderate-intensity physical activity a week or 75 min of intense activity a week (https://www.nhs.uk/live-well/exercise/exercise-guidelines/physical-activity-guidelines-for-adults-aged-19-to-64/ (accessed on 29 September 2021)). For the purposes of this analysis, the data were approximated to national PA recommendations by classifying participants as ‘physically active’ if they undertook any of the PA-related activities for at least 14 days per month.

The frequency of visits to IWs was used both as a plausible predictor of health outcomes and as an outcome variable. Visit frequency had five response levels: at least once a week, at least once a month, at least once a year, less frequently than once a year, or never.

### 2.3. Features of IWs

Following consultation with the Trust team and based on existing evidence, a list of IW features that were hypothesised to have an impact on each of the outcome measures was created (Table 1).

For each participant, the locality of the IW visits was determined based on the reported visits within the past 2 weeks of completing the survey. Visit status was classified as ‘urban’ if most visits were within urban settings; ‘rural’ if most of their visits were in rural areas; and ‘mixed’ if the participant visited both with the same frequency. Data on activities per type of IW (i.e., canals, rivers) were not included in the analysis to avoid introducing bias due to the high numbers of inconsistent or missing responses.

Additional grouping or dichotomisation of variables with many categories (e.g., visit frequency) was avoided for IW features as it could result in a loss of information on trending patterns in effect sizes. Trends in outcome effects could provide useful information on levels of IW usage for public health recommendations.

### 2.4. Covariates

Covariates that are known to influence LS and mental wellbeing were controlled for, including participants’ age, gender, education, employment, ethnicity, marital status, children (yes/no), limiting health problems, and self-reported health [25]. Given the high number of response levels and small number of observations in some categorical variables, categories were grouped together to increase statistical power. Furthermore, the analyses were adjusted to include two additional covariates that may have an impact on health and/or visitation rates; the value that participants placed on being outdoors (measured using a five-point Likert scale of enjoyment); and the value participants placed on being routinely active (five-point Likert scale of agreement).

### 2.5. Statistical Analyses

All statistical analyses were performed in STATA (version 17, StataCorp LLC, College Station, TX, USA). Both bivariate and multifactorial correlations between IW features and health outcomes (i.e., LS, wellbeing, and PA) were explored. In line with previous studies [26], LS was assumed to be cardinal to allow for the use of parametric models (i.e., ordinary least squares) that can be easily interpreted. Continuous variables were inspected for normality, and the results showed acceptable levels of skewness and kurtosis. No collinearity was found between covariates used in the multivariable models. Multiple imputation using the data augmentation algorithm was applied to the explanatory variables ‘visit frequency’ and ‘waterways encourage more exercise’ with levels of missingness at 12% and 10%, respectively. Data were assumed to be missing at random and regression analyses were performed using the imputed data.

ANOVA and *t*-tests were used to identify the IW features that are independently associated with firstly LS, and secondly wellbeing scores. The post hoc Bonferroni test was applied to ANOVA to correct for multiple comparisons. Significant results were used to inform the multi-factorial models.

Four multivariable regression models were developed to observe the combined effect of IW features on each of the four outcomes—LS, wellbeing, physical activity, and visit frequency. To account for the inherent complexity and broad range of IW features, selection of the models was based on combined information collected by the univariate analyses, existing evidence, and expert input from the Canal & River Trust partners. Known determinants of outcome measures were also included in the multivariable models. The models are presented in Appendix A, Table A4.

Ordinary least square (OLS) regression analysis was deemed appropriate for Model 1 and Model 2 since both LS and SWEMWBS were treated as continuous variables. Physical activity was binary categorical (active/non-active), and visit frequency was ordered categorical; therefore, logistic and ordered logistic regression were used in Model 3 and Model 4, respectively. In Model 4, visit frequency was regressed against other IW features and covariates. Since data on locality applied only to visits within the previous 2 weeks, inclusion of this variable in the factor list decreased the sample size in the model. To observe differences in the results and test the robustness of findings, all multivariable models were run with and without ‘locality’.

### 2.6. Subgroup Analysis

A subgroup analysis was conducted for Model 4 to observe variation in visit frequency by age using the following groups; 16–34, 35–54, 55–64, and 65+ years. Model 4 was applied to each age group and results were reported separately to observe deviations from the base case in visit frequency effects.

### 2.7. Travel-Cost Method

The travel-cost method (TCM), initially proposed by Hotelling (1947) and further developed by Clawson and Knetsch (2011) [22], was used to estimate user demand and infer the willingness to pay (WTP) for visiting the IWs. The TCM is based on the premise that, in the absence of a direct cost (user fee), the WTP for recreational sites can be derived from the travel costs and time associated with travelling to the site [27]. The TCM was performed for the whole sample and for different buffer zones of travel distance to waterways. Six buffer zones were selected based on the existing literature [28].

#### Unit Travel Cost Calculations and Buffer Zones Used in the TCM

For the TCM, unit costs of distance and time were calculated for participants’ visits to waterways. In the WEM survey, travel distances for each visit were calculated by and represented the straight-line distance from home postcode to IW postcode at the point of access based on the British coordinate system (https://epsg.io/27700 (accessed on 17 March 2021)). To account for multiple visits and site locations, an average travel distance from the home postcode to the IW postcode was calculated for each participant for all visits made in the 2 weeks prior to survey completion. The number of annual visits was estimated by scaling up the number of visits within the past 2 weeks. To avoid overestimation of costs, respondents who reported that they only travelled through IWs elsewhere were excluded from the analysis. Based on evidence from the National Travel Survey 2019 (NTS) [29], travel distances under 1 mile were considered ‘walking distance’, and for distances longer than 1 mile it was assumed a car was the preferred mode of travel. Participants’ travel time was calculated by combining the average distance with speed; an assumed speed of 3 mph was used for walking distances, and a speed of 25 mph (mean car speed in NTS [29]) was used for longer distances. The average UK mileage cost of 40 p/mile was assigned to ‘non-walking’ trips. The Department of Transport (WEBTAG) [30] unit cost of non-work time of 5.13 GBP/h was used to calculate travel time costs. The total travel cost was then derived from the total trip cost and the time cost for each individual.

The TCM was performed for the whole sample and for different buffer zones around the waterways. Buffer zones were selected based on travel distance to waterways as follows [28]:

Zone 1: ≤1 mile (walking distance)
Zone 2: >1 mile to 3 mile
Zone 3: >3 mile to 5 mile
Zone 4: >5 mile to 10 mile
Zone 5: >10 mile to 20 mile
Zone 6: >20 mile

A zero-truncated negative binomial count model was applied to regress the number of visits against the total travel cost (time costs plus trip costs) and covariates and to graph the demand curves. This estimation accounts for both the impossibility of a zero and the over-dispersion evidenced in the visits data. Individual socioeconomic characteristics were used as covariates in the model. As a first step, the demand model was applied to the whole sample, and then to each buffer zone, and the results were combined graphically in a demand curve. The demand curve presents different quantities of a commodity that are requested at different prices (or costs) of the same commodity [31] and thus the number of visits people would make at different travel cost prices. From this demand function, we can derive the average travel cost, which represents the average visitor’s (WTP) to visit the waterways.

Secondly, the combined effects of IW features and individual socioeconomic characteristics on the travel costs were explored in a separate regression model. This analysis was performed on the whole sample.

## 3. Results

### 3.1. Sample Characteristics

The sample characteristics are presented in Table 2. The sample included over 21,000 IW users with a mean age of 48 years. Approximately half the sample were female (51%), and the majority were white (86%), and educated at A/O level (47%). Similar to the national average [32], more than half of the sample were married, co-habiting, or in a civil partnership, and 79% had no dependent children.

Most participants were physically active (76%) or enjoyed having an active lifestyle (53%), whilst few reported a limiting health problem. Mean life satisfaction in the sample was 6.28, which is lower than the national average, and the mean wellbeing score was 23, indicating the absence of mental disorders such as anxiety or depression.

Regarding IW usage (Table 3), most visits were of a recreational/leisure nature and took place in urban settings. In most cases, visit frequency was between once a week and once a month. Fifty-eight per cent of participants reported at least one visit in the past 3 months. Visit satisfaction was high in the sample and most participants lived greater than 1km from an IW. Although most visits were not classified as related to PA, there was a general agreement amongst participants that IWs encouraged more exercise than usual.

### 3.2. Bivariate Associations

Higher mean LS was independently associated with use of IWs for recreation/leisure (mean LS = 6.51, SD = 2.26), visiting most days (mean LS = 6.76, SD = 2.52), rural visits (mean LS = 6.62, SD = 2.22), high visit satisfaction (mean LS = 6.79, SD = 2.39), mostly PA-related visits in past 3 months (LS = 6.65, SD = 2.22), and perceptions that IWs increased PA levels (mean LS = 6.7, SD = 2.46). The between-group differences in mean LS scores were significant at *p* = 0.05 level.

Higher mean wellbeing scores were observed amongst participants that used the IWs for recreation/leisure, reported high visit satisfaction, and perceived that waterways encouraged more PA. Higher SWEMSWB scores varied from 22.87 (SD = 4.37) to 24.13 (SD = 5.05).

### 3.3. Multivariable Associations

Full regression results are presented in Table A1 and Table A2 in Appendix A. Here, we report the statistically significant findings defined as a *p* value less than 0.05.

#### 3.3.1. Life Satisfaction

Higher LS was reported amongst participants who were retired (β = 0.396, 95%CI; 0.251, 0.540), male (β = 0.101, 95%CI; 0.022, 0.180), or those with better overall health (β = 0.047, 95%CI; 0.044, 0.049). Use of waterways for travel purposes or for recreation/leisure, and visits to rural settings were predictors of higher LS.

Conversely, lower LS levels were observed for participants who were unemployed (β = −0.303, 95%CI; −0.0429, −0.177), not married, educated below College-level (β = −0.108, 95%CI; −0.190, −0.024 for A-level), and those that do not enjoy being active (gradient effect in coefficients). Less frequent visits, low satisfaction from visits, and perceptions that waterways do not encourage more exercise than usual also had a negative effect on LS levels, which varied proportionally to the response levels of these variables.

#### 3.3.2. Mental Wellbeing

Results from Model 2 showed significantly higher wellbeing scores amongst older participants (β = 0.028, 95%CI; 0.006, 0.050), males (β = 0.620, 95%CI; 0.116, 1.12), and those with better overall health (β = 0.091, 95%CI; 0.079, 0.103). None of the IW features were found to significantly contribute to higher wellbeing. However, visiting for PA but living greater than 1km away had a combined negative effect (β = −0.867, 95%CI; −1.617, −0.1170) on participants’ wellbeing, alongside factors of low visit satisfaction, being unemployed (β = −0.792, 95%CI; −1.416, −0.167), being a part-time worker (β = −0.831, 95%CI; −1.44, −0.216), or tending to enjoy an active lifestyle (β = −0.593, 95%CI; −1.137, −0.050).

#### 3.3.3. Physical Activity

Model 3 aligns with well-known physical activity patterns reported in the literature. Use of waterways for recreational purposes increased the odds of being active by 1.2 times (95%CI; 1.005, 1.540). The likelihood of being active was lower when participants were older or had a limiting health problem. There was a pattern of decreasing odds of engaging in PA as participants visited less frequently, with the odds ratio (OR) varying from OR = 0.49 (95%CI; 0.305, 0.792) for visiting once a fortnight to OR = 0.28 (95%CI; 0.166, 0.473) for visiting less frequently than once a year.

#### 3.3.4. Visit Frequency

Frequent visits were significantly associated with accessing other places through waterways (OR = 1.97, 95%CI; 1.753, 2.223) and living within 1km of the IW (OR = 1.99, 95%CI; 1.808, 2.195). Males (OR = 1.37, 95%CI; 1.268, 1.474) and those who used IWs for exercise (OR = 1.31, 95%CI; 1.175, 1.453) were more likely to visit regularly. Interestingly, the positive effect on visit frequency was higher amongst participants with a limiting health problem (severe problems: OR = 1.77, 95%CI; 1.529, 2.057) compared to those with better overall health (OR = 1.00, 95%CI; 1.004, 1.008).

Significantly lower visit frequency was observed among respondents who were of older age (OR = 0.98) or lower education (A levels: OR = 0.88), retired (OR = 0.86), or unemployed (OR = 0.74). Visit frequency was also lower among those who did not value an active lifestyle and those who did not feel that IWs encouraged more PA. Finally, lower visit satisfaction resulted in proportionally less frequent visits. Despite the overall benefit from rural visits to LS that was observed in Model 1, participants reported less frequent visits to rural settings (OR = 0.79, 95%CI; 0.720, 0.859) than urban.

The factor variable ‘enjoy being outdoors’ was inconclusive in almost all models as the direction of effects remained constant irrespective of the response level. Therefore, regression results for this variable are not reported.

### 3.4. Subgroup Analysis

Results from the subgroup analysis are presented in Table A3 (Appendix A). Compared to the whole sample, some predictors of visit frequency were shown to be sensitive to age while for others, the effects remained unchanged. Differences in the direction, significance and magnitude of effects are presented below.

In the younger age group (16–34), participants with children tended to visit more frequently (OR = 1.16, 95%CI; 1.000, 1.343) compared to those without children. For participants aged over 35 years, PA predicted higher visit frequency, and its effect followed an increasing trend with age (16–34: OR = 1.14; 35–54: OR = 1.33; 55–64: OR = 1.53). Being of Asian or Black/Other ethnic origin was shown to have a negative impact on visit frequency within the age categories 35–54 years and 55–64 years. Whilst a major limiting health problem encouraged more frequent visits up to age 54 years, it became a barrier for older participants in the 55–64 age group (OR= 0.58, 95%CI; 0.1360, 0.3627) but did not impact visit frequency in the 65+ age group.

Lastly, the magnitude of effect for living close to IWs progressively increased from 1.49 (95%CI; 1.2498, 1.7937) in the age group 16–34 years to 2.97 (95%CI; 2.2826, 3.8670) for ages 65 and above (35–54: OR = 1.93, 55–64: OR = 2.77). This upward trend in the prediction of visit frequency highlights that distance can become a preventing factor of IW visits as people grow older.

### 3.5. Travel Costs and Users’ Demand

**Figure 1:**
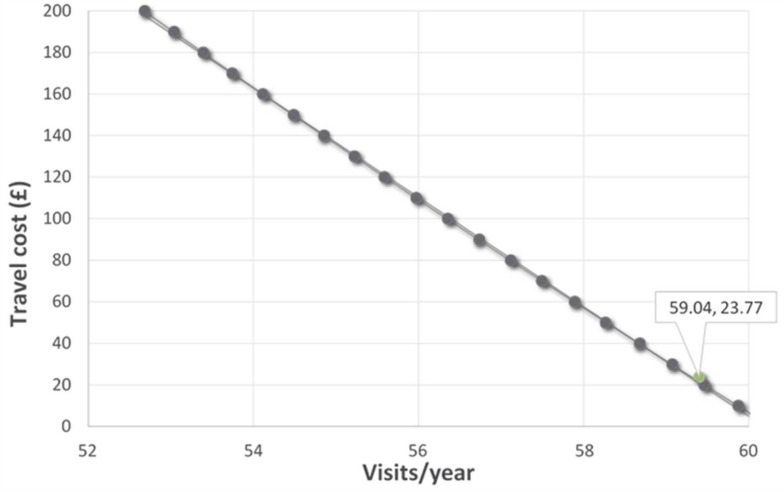
Whole sample demand curve and model estimates.

The mean travel cost per round trip in the sample was GBP 23.77 (SD: GBP 42.95), which shows the average WTP for visiting the waterways. On average, users made approximately 60 visits per year (SD: 56.87) or 5 visits per month to IWs. Based on the demand model for the whole sample, there was a 1% decrease in visits for every additional 1 GBP in total cost to visit (IRR = 0.99). There is a negative linear association indicating that the number of visits decreased as the travel cost increased and this is typical of a downward-sloping line as shown in Figure 1.

**Figure A1:**
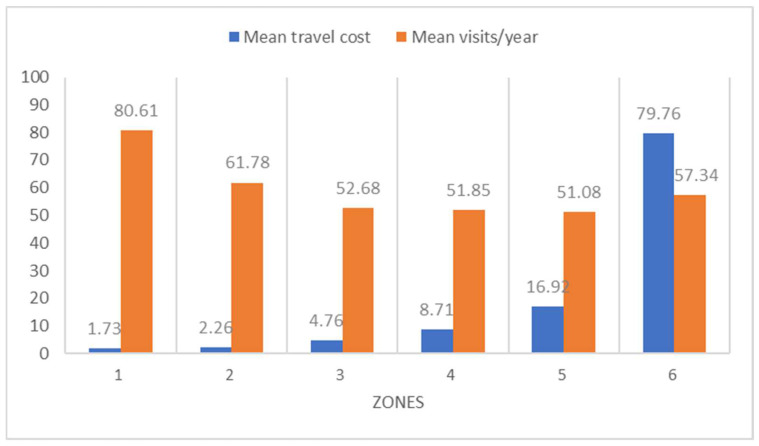
Mean travel costs and mean number of annual visits per buffer zone.

As expected, the results by buffer zone showed variation in costs and number of visits. The highest mean annual number of visits was 81 (SD: 81.55) for Zone 1 followed by lower visits to other zones in descending order, as shown in Appendix A, Figure A1.

**Figure 2:**
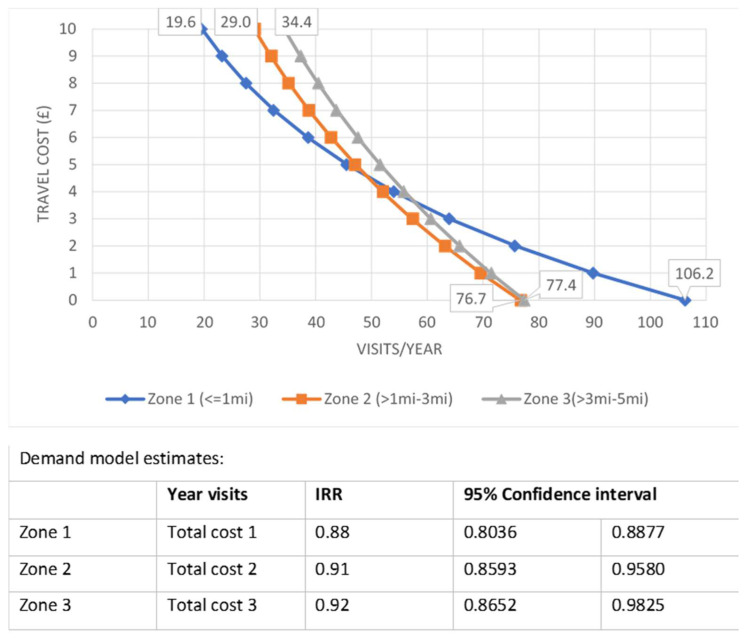
Demand curve and model estimates for Zones 1, 2, and 3.

Total cost had a statistically significant effect on the number of visits only for short distances defined as ≤5 mi, so the demand curves for Zones 1, 2, and 3 were calculated and combined graphically (Figure 2). All three curves followed a downward slope, which indicates a decreasing demand for visiting the waterways as the travel cost increases, irrespective of buffer zone. However, a higher drop rate of visits was observed for Zone 1 compared to Zones 2 and 3. As shown in Figure 2, for walking distances (Zone 1) there were 86 fewer visits for a GBP 10 increase in costs, while the reduction was almost half (43 visits) in Zone 3. The baseline assumption that all trips under 1 mile were within walking distance was tested in a sensitivity analysis where 0.5 mi and 0.2 mi were used as cut-off points for Zone 1. No substantial differences were observed in the demand curves compared to the base case, and therefore, sensitivity analysis results were not reported. Consequently, demand for longer trips appears more inelastic, and thus less sensitive to changes in travel cost compared to shorter distances.

In the second part of the TCM, a linear regression model was applied to estimate the effects on travel cost from IW features and individual socioeconomic factors. As shown in Table 4, participants who were out of employment or from an Asian origin incurred higher travel costs when visiting the IWs. Lower travel costs were incurred by participants who were accessing the IW site to be physically active, had children, or were within an older age group. This concurs with the wider evidence that people prefer local settings to exercise and with the earlier findings from this study that older people visit IWs more frequently if they are within close proximity to their household.

## 4. Discussion

The aim of this study was to generate evidence on the associations between IW use and three health outcomes: life satisfaction, mental wellbeing, and physical activity. Secondary aims were to scrutinise determinants of visit frequency and to estimate travel costs to observe trends in user demand for IWs. The findings showed that frequent visits to IWs and use for recreational/leisure purposes were both beneficial to LS and PA levels. Rural visits had a positive effect on LS, compared to urban ones, while none of the IW features were found to increase mental wellbeing. Low visit satisfaction had a negative impact on LS and mental wellbeing. Visit frequency was mostly influenced by individual characteristics and purpose of visit, including visits for exercise. As theory predicts, user demand for visiting the waterways was inversely associated to travel costs, and the results by buffer zone showed greater elasticity of demand for shorter distances.

Within the sample, most participants were physically active and agreed that waterways can increase PA levels. PA had a synergistic effect in the association between IW usage and health in two instances; firstly, a positive effect on LS was detected for participants who considered waterways as a means to increase their PA, and use of waterways for recreation/sport had a significant positive effect on LS levels. Secondly, PA-related visits were a predictor of more frequent visits to IWs, which successively resulted in higher levels of LS. Regular PA has long been associated with higher levels of LS, and evidence demonstrates improvements in mental health and wellbeing from engaging in exercise in natural environments [33,34]. In our sample, there is a reciprocal relationship between PA and IW visits, which resulted in significant psychological benefits for participants. This twofold role of natural environments in both physical health and mental wellbeing was also found in a review of previous studies [35] that highlighted the importance of promoting accessible blue spaces as a vehicle to ameliorate population health. Additionally, investment in regeneration and maintenance projects could increase demand for visits as people begin to realise the benefits of IW and gain more satisfaction from visiting [36].

Accessibility was one of the key factors contributing to more frequent visits and higher LS in the study. Living close to a waterway stretch increased visits with effects following an upward trend with age. Even though rural settings were considered more beneficial to LS than urban locations, rural environments were visited less frequently by users, possibly due to long travel distances and limited accessibility. Close proximity to IW may contribute to higher participation in PA if waterways are integrated within the urban web, which has implications for urban planning policies. UK guidelines recommend a provisional distance of 300m (5mins) from households to green/blue spaces for people living in urban settings; however, this is less feasible in cities with high structural density [37].

Thematic analysis of potential barriers and enablers for users’ engagement with waterways in the WEM survey revealed that lack of information on local sites and accessibility issues (e.g., lack of suitable paths) were the most reported barriers to visiting the waterways. Information campaigns could be used as a means to raise visitor awareness about IWs, and improved transport links were suggested for encouraging waterway usage. In this study, travelling through waterways to access other places increased visit frequency and LS, which highlights the need for shifting policy towards using IWs to achieve ‘active’ ways of travel (i.e., walking and cycling) as a means of providing health benefits to adults [38] and children [39].

In our study, inequalities in IW usage and travel costs were evident regarding users’ ethnicity, gender, age, education, and employment. Similar findings from the WHO European region concluded that low socioeconomic position was associated with a lack of well-maintained, available and accessible natural resources both on the neighbourhood and individual levels [20]. Likewise, socioeconomic and demographic factors are often considered confounders in the association between health and natural environments with greater protective effects observed for deprived populations [40]. In addition to health inequalities, unequal access can be translated to longer travel distances and proportionately higher costs for specific population subgroups. Future policies to address these health disparities should prioritise investment in high-quality blue space that is available and accessible to disadvantaged groups as a means of closing the gap in health outcomes.

### Strengths and Limitations

A strength of this study is the large sample size of 21,000 respondents. Participants were recruited by proprietary methods based on known characteristics, and the sample was widely representative of the general population. Additionally, a variety of information was collected that related to aspects of waterway usage, health and wellbeing, lifestyle, and individual characteristics. The diversity of data enabled a comprehensive analysis of multifactorial associations between outcome variables and combinations of IW features and sociodemographic characteristics.

Regarding limitations, the self-reported nature of the outcomes may have been a weakness. Response bias is a common issue in self-reported measures of wellbeing as such data are susceptible to over-reporting, mainly due to factors relating to individuals’ mood and social desirability [41]. Therefore, we acknowledge that the interpretation of results should be made with caution. It is advised that objective alternative measures should also be included, where possible, to diversify the data. Furthermore, data on the use of waterways were collected retrospectively, which may have introduced added uncertainty in participants’ responses largely attributed to recall bias. An additional limitation was that data on visits to different types of waterways (i.e., canal or river) was omitted from the analysis due to the low quality of responses; thus, no differential effects based on IW type could be shown in the results. Another limitation was that, in the absence of other data, a set of assumptions was applied to transform reported daily PA based on PA guidelines (150 min per week) and, secondly, estimate annual visits alongside subsequent travel costs by using a 2-week reporting period as a representation of the average. According to similar recreational visit surveys [42,43], recall periods of up to 4 weeks are appropriate when other data are not available. Furthermore, our sample was limited to existing users of waterways, who may follow a more active lifestyle or be more willing to participate in surveys.

## 5. Conclusions

Frequent use of IWs for recreation or leisure is associated with improved physical health and mental wellbeing in the population. Future policies could enhance accessibility and use of waterways to alleviate health-related inequalities across population groups.

### Research and Policy Recommendations

- Frequent use of IWs for recreation or leisure is beneficial to life satisfaction and physical activity in the population.
- Improved accessibility of IWs contributes to more frequent visits and higher LS.
- Future investment in regeneration and maintenance projects is needed for the provision of accessible blue spaces that are integrated into the urban web.
- There is evidence of existing inequalities in how IWs are accessed and used based on ethnicity, gender, age, and employment. Policy design to ameliorate health inequalities linked to unequal access and use of IWs could prioritise interventions that target disadvantaged populations.
- To inform investment on IW interventions given existing public budget constraints, further research is needed on the cost-effectiveness of alternative approaches to improve accessibility.

## Figures and Tables

**Table 1:** Features of waterway usage.

Waterway Feature	Response Levels *
Type of IW use	To access greenspace (r) Recreation, leisure Travel purposes
Locality of visits **	Urban (r) Rural Mixed
PA most important reason for visit	Yes No (r)
Visit frequency	Varying frequency (most days/a few times a week/around once a fortnight/ around once a month/at least once a week/between every 3 months and once a year/between once a month and every 3 months/less frequently than once a year/never) Most days (r)
Proximity to IW	Live within 1 km of IW Live >1 km from IW (r)
Visit satisfaction	Five-point Likert scale (very satisfied, quite satisfied, Neither satisfied nor dissatisfied, Quite dissatisfied, very dissatisfied Very satisfied (r)
Waterways encourage more exercise	Five-point Likert scale (strongly agree/tend to agree/neither agree nor disagree/tend to disagree/strongly disagree) Strongly agree (r)

* The (r) indicates the ‘reference category’. ** Locality was only asked of participants who visited within the past 2 weeks.

**Table 2:** Sample characteristics.

Variable	Frequency (n)	Mean (SD) or %	National Average Values (Mean or %)
Age	21,537	47.56 (17.66)	40.3
Gender			
Female	11,192	51.97	51
Male	10,200	47.36	49
Non-binary	52	0.24	-
Own term	7	0.03	-
Prefer not to say	36	0.17	-
Transgender	49	0.23	-
Education			
A level/O level	10,146	47.11	40.9
College/Higher degree	7687	35.69	27
Professional	2224	10.33	9.1
No formal	1161	5.39	23
Prefer not to say	319	1.48	-
Employment			
Full-time	8965	41.63	48.1
Part-time	2710	12.58	13.7
Retired	5146	23.89	13.8
Unemployed (incl. informal carers and students)	4370	20.29	24.3
Prefer not to say	346	1.61	-
Ethnicity			
White	19,316	89.69	86
Asian	1150	5.34	7.5
Black/Any other ethnic group	820	3.81	6.5
Prefer not to say	251	1.17	-
Marital status			
Married/Civil partnership	12,101	56.19	50.9
Not married	8285	38.47	40.7
Widowed	756	3.51	8.4
Prefer not to say	395	1.83	*-*
Children			
Yes	4497	20.88	29
No	17,040	79.12	71
Limiting health problem			
Yes, limited a lot	2169	10.07	18
Yes, limited a little	4478	20.79	
No	14,532	67.47	82
Prefer not to say	358	1.66	-
Enjoy being active			
Neither agree nor disagree	3816	17.72	-
Strongly agree	4117	19.12	-
Strongly disagree	3724	17.29	-
Tend to agree	7219	33.52	-
Tend to disagree	2661	12.36	-
Enjoy being outdoors			
Neither agree nor disagree	5266	24.45	-
Strongly agree	3357	15.59	-
Strongly disagree	1086	5.04	-
Tend to agree	8474	39.35	-
Tend to disagree	3354	15.57	-
Life satisfaction (0—lowest to 10—highest) *	18,982	6.28 (2.47)	7.4
SWEMWB score (7—lowest to 35—highest positive wellbeing) *	2433	22.63 (4.54)	24.6
Overall health	21,537	65.6 (22.95)	-
Physically active (≥14 days in the past month) *			
Yes	5723	76.32	-
No	1776	23.68	-

* Life satisfaction, SWEMWB scale, and physical activity were asked in different waves of the survey—a complete case analysis was performed.

**Table 3:** IW Usage.

Variable	Frequency (n)	Percentage (%)
Type of use		
For travel purposes (to go somewhere else)	2370	19.85
Recreation, sport, tourism	6619	55.45
To access greenspace	2948	24.7
Frequency of visits *		
A few times a week	1542	8.16
Around once a fortnight	1785	9.44
Around once a month	2167	11.46
At least once week	2016	10.66
Between every 3 months and once a year	2797	14.8
Between once a month and every 3 months	1850	9.79
I have never visited or used this type	1437	7.6
Less frequently than once a year	4339	22.95
Most days	972	5.14
Last visit within past 3 months	11,937	58
Live within 1km of a canal or river	2971	13.79
Locality of visits **		
Urban	6315	64.95
Rural	2681	27.57
Mixed	727	7.48
PA most important reason for visit		
Yes	1395	11.7
No	10,542	88.3
Waterways increased PA levels *		
Neither agree nor disagree	2763	25.76
Strongly agree	2156	20.1
Strongly disagree	137	1.28
Tend to agree	5171	48.21
Tend to disagree	498	4.64
Visit satisfaction **		
Neither satisfied nor dissatisfied	1066	8.97
Quite dissatisfied	419	3.52
Quite satisfied	6237	52.46
Very dissatisfied	251	2.11
Very satisfied	3917	32.94

* Missing values were imputed for the analysis. ** Missing values < 5%.

**Table 4:** Regression results of travel cost predictors.

Variables	β-Coef (95% Confidence Interval)
Age	−0.01 (−0.016, −0.008)
Asian	0.32 (0.108, 0.535)
Rural sites	0.54 (0.446, 0.646)
Visit frequency	
Around once a month	0.24 (0.038, 0.451)
Between once a month and every 3 months	0.44 (0.214, 0.662)
Between every 3 months and once a year	0.68 (0.445, 0.912)
Less frequently than once a year	0.89 (0.637, 1.146)
Retired	0.16 (0.006, 0.311)
Widowed	0.30 (0.003, 0.609)
A level/O level	−0.15 (−0.248, −0.050)
Children-Yes	0.21 (−0.318, −0.097)
Live within 1 km of a canal/river	−0.49 (−0.609, −0.370)
Recreation/tourism	0.24 (0.140, 0.340)
Reason for visit-PA	−0.32 (−0.452, −0.193)
Very dissatisfied user	0.38 (0.059, 0.702)

## Data Availability

The data that support the findings of this study are available from Canal & River Trust, but restrictions apply to the availability of these data, which were used under license for the current study, and so are not publicly available.

## Appendix A

**Table A1:** Multivariable models of association with control variables.

		Model 1—Life Satisfaction			Model 2—SWEMWBS			Model 3—Physical Activity			Model 4—Visit Frequency		
Independent Variables (Predictors)	Reference Category	Coefficient	95% CI		Coefficient	95% CI		Estimates (OR)	95% CI		Estimates (OR)	95% CI	
constant		4.1373	3.6946	4.5800	16.4889	14.5928	18.3850	18.8332	9.0246	39.3024			
Age		−0.0355 **	−0.0507	−0.0203	0.0283 **	0.0092	0.0475	0.9828 **	0.9747	0.9909	0.9891 **	0.9859	0.9922
Age squared		0.0005 **	0.0003	0.0007									
Gender	Female												
Male		0.1013 **	0.0221	0.1805	0.5123 *	0.0705	0.9540	1.1552	0.9457	1.4111	1.3714 **	1.2721	1.4784
Other		−0.0648	−0.6150	0.4855	−1.6636	−3.8537	0.5266	1.0000			1.8255 **	1.1326	2.9422
Employment	Full-time												
Part-time		−0.0968	−0.2162	0.0225	−0.8314 **	−1.4460	−0.2168	1.1375	0.8331	1.5533	0.9395	0.8414	1.0489
Retired		0.3963 **	0.2519	0.5407	−0.0735	−0.7558	0.6087	1.1715	0.8583	1.5988	0.8549 **	0.7586	0.9634
Unemployed		−0.3033 **	−0.4293	−0.1774	−0.7920 **	−1.4164	−0.1677	1.0236	0.7770	1.3485	0.7372 **	0.6604	0.8230
Prefer not to say		−0.6644 **	−1.0906	−0.2383	−1.4596	−3.5072	0.5879	1.6761	0.5881	4.7767	0.6960	0.4630	1.0463
Education	College/Higher												
A level/O level		−0.1078 **	−0.1907	−0.0249	−0.1477	−0.5907	0.2953	0.9676	0.7898	1.1855	0.8739 **	0.8092	0.9438
Professional		−0.0856	−0.2166	0.0454	−0.4449	−1.0471	0.1573	0.9953	0.7279	1.3610	0.9592	0.8524	1.0794
No formal		0.1392	−0.0822	0.3607	1.2859	−0.1312	2.7030	0.9530	0.5564	1.6323	0.8568	0.6882	1.0667
Prefer not to say		0.3574	−0.1199	0.8346	−1.8594	−3.8255	0.1067	0.9315	0.2545	3.4092	1.3473	0.8366	2.1699
Ethnicity	White												
Asian		0.0974	−0.0579	0.2527	0.1486	−0.9149	1.2120	1.3988	0.9059	2.1599	0.8709	0.7341	1.0332
Black/Any other ethnic group		−0.0899	−0.2870	0.1073	−0.7267	−1.6940	0.2407	0.8150	0.5110	1.2999	1.0841	0.8951	1.3130
Prefer not to say		0.2468	−0.2127	0.7063	−1.6983	−3.6631	0.2666	0.9571	0.3739	2.4500	1.1339	0.7218	1.7814
Marital status	Married												
Not married		−0.5775 **	−0.6692	−0.4858	−0.4394	−0.9097	0.0309	1.2305	0.9872	1.5338	-	-	-
Widowed		−0.6254 **	−0.8947	−0.3562	−1.0018	−2.3684	0.3648	1.2030	0.6564	2.2046	-	-	-
Prefer not to say		−0.4651 **	−0.8531	−0.0770	−0.4770	−2.0331	1.0791	1.6160	0.6301	4.1446	-	-	-
Children	No										-	-	-
Yes		0.0849	−0.0095	0.1793	−0.2159	−0.7367	0.3049	1.0376	0.8169	1.3180	-	-	-
Marital status and children	Married and No children												
Married and children		-	-	-	-	-	-	-	-	-	1.0125	0.9116	1.1246
Not married and no children		-	-	-	-	-	-	-	-	-	0.9066 *	0.8278	0.9930
Not married and children		-	-	-	-	-	-	-	-	-	1.0574	0.8942	1.2503
Widowed and no children		-	-	-	-	-	-	-	-	-	1.3100 *	1.0439	1.6441
Widowed and children		-	-	-	-	-	-	-	-	-	1.2255	0.6328	2.3731
Prefer not to say and no children		-	-	-	-	-	-	-	-	-	1.0314	0.7064	1.5060
Prefer not to say and children		-	-	-	-	-	-	-	-	-	0.5944	0.2300	1.5358
Limiting health problem	Not limited												
Prefer not to say		0.2488	−0.1383	0.6359	1.2437	−1.0952	3.5827	0.6178	0.2623	1.4554	1.2744	0.8534	1.9032
Yes, limited a little		−0.0320	−0.1374	0.0735	0.0510	−0.4675	0.5696	0.8391	0.6628	1.0622	1.4401 **	1.3086	1.5849
Yes, limited a lot		−0.1331	−0.2964	0.0302	0.7895	−0.0951	1.6741	0.4222 **	0.3119	0.5715	1.7724 **	1.5281	2.0558
Overall health		0.0473 **	0.0449	0.0496	0.0914 **	0.0792	0.1037	1.0099 **	1.0059	1.0140	1.0064 **	1.0046	1.0082

* *p*-value ≤ 0.05, ** *p*-value ≤ 0.01. 95% CI: 95% Confidence interval.

**Table A2:** Multivariable models of association with IW features.

		Model 1—Life Satisfaction			Model 2—SWEMWBS			Model 3—Physical Activity			Model 4—Visit Frequency		
Independent Variables (Predictors)	Reference Category	Coefficient	95% CI		Coefficient	95% CI		Estimates (OR)	95% CI		Estimates (OR)	95% CI	
Canal proximity and PA most important reason	Do not live within 1km of a canal or river and PA-no												
DO NOT live within 1 km of a canal or river and PA-yes		-	-	-	−0.8673 *	−1.6176	−0.1171	-	-	-	-	-	-
Live within 1 km of a canal or river and PA-no		-	-	-	−0.4016	−0.8914	0.0883	-	-	-	-	-	-
Live within 1 km of a canal or river and PA-yes		-	-	-	0.3535	−0.9107	1.6177	-	-	-	-	-	-
Canal proximity	Do not live within 1km of a canal or river												
*Live within 1 km of a canal or river*		−0.0651	−0.1661	0.0359	-		-	0.8918	0.6896	1.1532	1.9972 **	1.8124	2.2009
Frequency of visits	Most days												
Few days a week		−0.0184	−0.1878	0.1511	−0.9924	−2.1167	0.1320	0.8477	0.5098	1.4096	-	-	-
At least once a week		−0.1922 *	−0.3601	−0.0243	−0.3058	−1.4035	0.7919	0.6675	0.4147	1.0744	-	-	-
Around once a fortnight		−0.2351 **	−0.4073	−0.0630	−0.6298	−1.7554	0.4958	0.4917 **	0.3049	0.7927	-	-	-
Around once a month		−0.3148 **	−0.4866	−0.1430	−0.5754	−1.6736	0.5227	0.4095 **	0.2564	0.6541	-	-	-
Between once a month and every 3 months		−0.3277 **	−0.5113	−0.1440	−0.8933	−2.0273	0.2407	0.3884 **	0.2381	0.6338	-	-	-
Between every 3 months and once a year		−0.3390 **	−0.5312	−0.1467	−0.3803	−1.6408	0.8803	0.3470 **	0.2090	0.5760	-	-	-
Less frequently than once a year		−0.2840 **	−0.5074	−0.0606	−0.6887	−1.9745	0.5971	0.2807 **	0.1665	0.4733	-	-	-
I have never visited or used		−0.0315	−0.5118	0.4487	0.0921	−2.2520	2.4363	0.5280	0.1308	2.1322	-	-	-
Type of use	To access greenspace												
For travel purposes		0.1963 **	0.0748	0.3178	0.1101	−0.6037	0.8239	1.1475	0.8647	1.5229	1.9778 **	1.7561	2.2276
Recreation, sport, tourism, leisure activities		0.1203 **	0.0285	0.2122	−0.0259	−0.5257	0.4738	1.2440 *	1.0047	1.5402	0.9834	0.9066	1.0667
PA most important reason	No												
Yes		−0.0440	−0.1533	0.0652	-	-		1.1473	0.8677	1.5170	1.3063 **	1.1751	1.4521
Enjoy being active	Strongly agree												
Tend to agree		−0.0845	−0.1868	0.0178	−0.5936 *	−1.1372	−0.0500	-	-	-	1.0248	0.9299	1.1293
Neither agree nor disagree		−0.2283 **	−0.3621	−0.0945	−0.2091	−1.0141	0.5958	-	-	-	0.8723 *	0.7707	0.9873
Tend to disagree		−0.2458 **	−0.3908	−0.1007	−0.3217	−1.0197	0.3763	-	-	-	0.8831	0.7740	1.0076
Strongly disagree		−0.2588 **	−0.4048	−0.1128	−0.2965	−0.9996	0.4067	-	-	-	0.7859 **	0.6898	0.8955
Waterways increased PA levels	Strongly agree												
Tend to agree		−0.0254	−0.1329	0.0820	−0.0853	−0.8003	0.6296	0.9368	0.7090	1.2378	0.7058 **	0.6373	0.7816
Neither agree nor disagree		−0.1118	−0.2390	0.0153	−0.1290	−0.8804	0.6225	0.9318	0.6824	1.2724	0.4578 **	0.4064	0.5158
Tend to disagree		−0.2380 *	−0.4559	−0.0201	0.5575	−0.9353	2.0503	0.6732	0.4411	1.0274	0.3372 **	0.2777	0.4095
Strongly disagree		−0.3143	−0.7095	0.0809	−1.0009	−3.7254	1.7237	0.8079	0.3607	1.8093	0.1643 **	0.1092	0.2472
Enjoy being outdoors	Strongly agree												
Tend to agree		0.2110 **	0.0986	0.3235	−0.1958	−0.8914	0.4997	0.5782 **	0.4543	0.7359	0.7646 **	0.6908	0.8462
Neither agree nor disagree		0.3476 **	0.2197	0.4756	0.8849 *	0.1232	1.6465	0.3668 **	0.2719	0.4949	0.7685 **	0.6823	0.8656
Tend to disagree		0.6201 **	0.4849	0.7554	1.9173 **	1.1286	2.7060	0.3341 **	0.2238	0.4989	0.7204 **	0.6356	0.8165
Strongly disagree		0.9120 **	0.7098	1.1142	3.5796 **	2.3968	4.7624	0.2490 **	0.1468	0.4223	1.1127	0.9145	1.3537
Visit satisfaction	Very satisfied												
Quite satisfied		−0.1894 **	−0.2768	−0.1020	−1.0571 **	−1.5503	−0.5638	0.9602	0.7684	1.1999	0.7995 **	0.7376	0.8667
Neither satisfied nor dissatisfied		−0.4093 **	−0.5688	−0.2498	−1.6968 **	−2.4753	−0.9184	1.2078	0.8460	1.7243	0.5933 **	0.5068	0.6947
Quite dissatisfied		−0.4225 **	−0.6375	−0.2074	−0.4766	−1.4980	0.5448	1.2209	0.7566	1.9701	0.6700 **	0.5570	0.8058
Very dissatisfied		0.0034	−0.2689	0.2758	1.0576	−0.5835	2.6987	1.1469	0.6252	2.1040	1.0901	0.8380	1.4180
Locality ^1^	Urban												
Rural		0.1049 *	0.0125	0.1973	-	-		-	-	-	0.7868 **	0.7204	0.8593
Mixed		0.0711	−0.0862	0.2285	-	-		-	-	-	0.9804	0.8497	1.1311

^1^ Regression results are presented excluding locality. The coefficient of ‘locality’ was added only when the variable was significant in the subsequent regression analysis. * *p*-value ≤ 0.05, ** *p*-value ≤ 0.01. OR: Odds ratio, 95% CI: 95% Confidence interval.

**Table A3:** Results from subgroup analysis on visit frequency.

Age Group: 16–34				
Independent Variables	Reference	Estimates (OR) *	95% Conf. Interval	
Gender	Female			
Male		1.6049	1.3920	1.8504
Employment	Full-time			
Unemployed		0.7678	0.6511	0.9054
Children	No			
Yes		1.1593	1.0005	1.3432
Limiting health problem	No			
Yes, limited a little		2.1505	1.7786	2.6001
Yes, limited a lot		3.0043	2.3631	3.8196
Overall health		1.0071	1.0040	1.0103
Canal proximity	Do not live within 1km of a canal or river			
Live within 1 km of a canal or river		1.4972	1.2498	1.7937
Type of use	To access greenspace			
For travel purposes, only in order to get somewhere else (..)		1.8472	1.5082	2.2623
PA most important reason	No			
Yes		1.1468	0.9189	1.4313
Locality	Urban			
Rural		0.7743	0.6629	0.9043
Enjoy being active	Strongly agree			
Strongly disagree		0.6663	0.4630	0.9590
Waterways increased PA levels	Strongly agree			
Neither agree nor disagree		0.6021	0.4793	0.7563
Strongly disagree		0.3295	0.1552	0.6995
Tend to disagree		0.3984	0.2804	0.5659
Enjoy being outdoors	Strongly agree			
Tend to agree		0.8141	0.6826	0.9708
Tend to disagree		0.7643	0.6026	0.9693
Visit satisfaction	Very satisfied			
Neither satisfied nor dissatisfied		0.5557	0.4296	0.7188
Quite dissatisfied		0.5925	0.4057	0.8652
Quite satisfied		0.7128	0.6116	0.8307
**Age Group: 35–54**				
Gender	Female			
Male		1.1947	1.0266	1.3904
Other		2.7289	1.1735	6.3457
Employment	Full-time			
Prefer not to say		0.3618	0.1531	0.8548
**Age Group: 55–64**				
Ethnicity	White			
Asian		0.3095	0.1344	0.7251
Black/Any other ethnic group		0.4210	0.1860	1.0008
Limiting health problem	No			
Yes, limited a little		1.3299	1.0446	1.6931
Yes, limited a lot		0.5760	0.3627	0.9149
Canal proximity	Do not live within 1km of a canal or river			
Live within 1 km of a canal or river		2.7718	2.1494	3.5744
Type of use	To access greenspace			
For travel purposes, only in order to get somewhere else (..)		1.7645	1.2174	2.5573
PA most important reason	No			
Yes		1.5323	1.2049	1.9487
Waterways increased PA levels	Strongly agree			
Neither agree nor disagree		0.4429	0.3294	0.5955
Strongly disagree		0.2408	0.1081	0.5365
Tend to agree		0.6898	0.5342	0.8906
Tend to disagree		0.4486	0.2523	0.7978
Enjoy being outdoors	Strongly agree			
Neither agree nor disagree		0.7314	0.5447	0.982
Tend to agree		0.7034	0.5356	0.9239
Tend to disagree		0.6745	0.4892	0.9299
Visit satisfaction	Very satisfied			
Neither satisfied nor dissatisfied		0.3386	0.2134	0.5372
Quite dissatisfied		0.507	0.3304	0.7781
**Age Group:** **65+**				
Gender	Female			
Male		1.2309	1.0164	1.4906
Employment	Full-time			
Retired		0.6617	0.4827	0.9071
Canal proximity	Do not live within 1km of a canal or river			
Live within 1 km of a canal or river		2.971	2.2826	3.867
Type of use	To access greenspace			
For travel purposes, only in order to get somewhere else (..)		1.5914	1.0884	2.3268
PA most important reason	No			
Yes		1.4503	1.1282	1.8645
Waterways increased PA levels	Strongly agree			
Neither agree nor disagree		0.4079	0.3050	0.5456
Strongly disagree		0.1940	0.0706	0.5330
Tend to agree		0.7318	0.5607	0.9550
Tend to disagree		0.3085	0.1865	0.5105
Enjoy being outdoors	Strongly agree			
Neither agree nor disagree		0.7571	0.5421	1.0575
Strongly disagree		0.8381	0.5125	1.3705
Tend to agree		0.7757	0.5779	1.0412
Tend to disagree		0.7446	0.5395	1.0277
Visit satisfaction	Very satisfied			
Quite satisfied		0.7824	0.6386	0.9587

OR: Odds ratio; 95% CI: 95% Confidence interval; PA: physical activity. * All results are statistically significant at *p*-value ≤ 0.05.

**Table A4:** Multivariable models.

Dependent (Outcome) Variable	Independent Variables
Model 1—Ordinary Least Squares (OLS)	
Life Satisfaction	waterway features * and covariates
Model 2—Ordinary Least Squares (OLS)	
SWEMWB score	waterway features * and covariates
Model 3—Logistic regression	
Physical activity(binary outcome: 1 if >14 days per month of walking/cycling/water sports/other, and 0 otherwise)	waterway features * and covariates
Model 4—Ordered logistic regression	
Visit frequency	waterway features * and covariates

* All models were analysed with and without locality.

## References

[1] Bowler D.E., Buyung-Ali L.M., Knight T.M., Pullin A.S. (2010). A systematic review of evidence for the added benefits to health of exposure to natural environments. BMC Public Health.

[2] Pretty J., Peacock J., Hine R., Sellens M., South N., Griffin M. (2007). Green exercise in the UK countryside: Effects on health and psychological well-being, and implications for policy and planning. J. Environ. Plan. Manag..

[3] Shanahan D.F., Bush R., Gaston K.J., Lin B.B., Dean J., Barber E., Fuller R.A. (2016). Health Benefits from Nature Experiences Depend on Dose. Sci. Rep..

[4] De Vries S., van Dillen S.M., Groenewegen P.P., Spreeuwenberg P. (2013). Streetscape greenery and health: Stress, social cohesion and physical activity as mediators. Soc. Sci. Med..

[5] Taylor L., Hahs A.K., Hochuli D.F. (2018). Wellbeing and urban living: Nurtured by nature. Urban Ecosyst..

[6] White M.P., Alcock I., Wheeler B.W., Depledge M.H. (2013). Would you be happier living in a greener urban area? A fixed-effects analysis of panel data. Psychol. Sci..

[7] Thompson C.W., Roe J., Aspinall P., Mitchell R., Clow A., Miller D. (2012). More green space is linked to less stress in deprived communities: Evidence from salivary cortisol patterns. Landsc. Urban Plan..

[8] Beyer K.M., Kaltenbach A., Szabo A., Bogar S., Nieto F.J., Malecki K.M. (2014). Exposure to neighborhood green space and mental health: Evidence from the survey of the health of Wisconsin. Int. J. Environ. Res. Public Health.

[9] Reklaitiene R., Grazuleviciene R., Dedele A., Virviciute D., Vensloviene J., Tamosiunas A., Baceviciene M., Luksiene D., Sapranaviciute-Zabazlajeva L., Radisauskas R. (2014). The relationship of green space, depressive symptoms and perceived general health in urban population. Scand. J. Public Health.

[10] Cohen D.A., McKenzie T.L., Sehgal A., Williamson S., Golinelli D., Lurie N. (2007). Contribution of public parks to physical activity. Am. J. Public Health.

[11] Evenson K.R., Wen F., Hillier A., Cohen D.A. (2013). Assessing the contribution of parks to physical activity using global positioning system and accelerometry. Med. Sci. Sports Exerc..

[12] Veitch J., Ball K., Crawford D., Abbott G., Salmon J. (2013). Is park visitation associated with leisure-time and transportation physical activity?. Prev. Med..

[13] Richardson E.A., Pearce J., Mitchell R., Kingham S. (2013). Role of physical activity in the relationship between urban green space and health. Public Health.

[14] Thompson Coon J., Boddy K., Stein K., Whear R., Barton J., Depledge M.H. (2011). Does participating in physical activity in outdoor natural environments have a greater effect on physical and mental wellbeing than physical activity indoors? A systematic review. Environ. Sci. Technol..

[15] Mitchell R. (2013). Is physical activity in natural environments better for mental health than physical activity in other environments?. Soc. Sci. Med..

[16] Britton E., Kindermann G., Domegan C., Carlin C. (2018). Blue care: A systematic review of blue space interventions for health and wellbeing. Health Promot. Int..

[17] Gascon M., Triguero-Mas M., Martínez D., Dadvand P., Forns J., Plasència A., Nieuwenhuijsen M.J. (2015). Mental health benefits of long-term exposure to residential green and blue spaces: A systematic review. Int. J. Environ. Res. Public Health.

[18] Gascon M., Zijlema W., Vert C., White M.P., Nieuwenhuijsen M.J. (2017). Outdoor blue spaces, human health and well-being: A systematic review of quantitative studies. Int. J. Hyg. Environ. Health.

[19] Pasanen T.P., White M.P., Wheeler B.W., Garrett J.K., Elliott L.R. (2019). Neighbourhood blue space, health and wellbeing: The mediating role of different types of physical activity. Environ. Int..

[20] Schüle S.A., Hilz L.K., Dreger S., Bolte G. (2019). Social Inequalities in Environmental Resources of Green and Blue Spaces: A Review of Evidence in the WHO European Region. Int. J. Environ. Res. Public Health.

[21] O’Gorman S., Bann C., Caldwell V. (2010). The Benefits of Inland Waterways.

[22] Clawson M., Knetsch J.L. (2011). Economics of Outdoor Recreation.

[23] Diener E. (2000). Subjective well-being. The science of happiness and a proposal for a national index. Am. Psychol..

[24] (2008). Short Warwick Edinburgh Mental Wellbeing Scale (SWEMWBS).

[25] Dolan P., Peasgood T., White M. (2008). Do we really know what makes us happy A review of the economic literature on the factors associated with subjective well-being. J. Econ. Psychol..

[26] Ferrer-i-Carbonell A., Frijters P. (2004). How Important Is Methodology for the Estimates of the Determinants of Happiness?. Econ. J..

[27] Trice A.H., Wood S.E. (1958). Measurement of Recreation Benefits. Land Econ..

[28] Wheeler B.W., White M., Stahl-Timmins W., Depledge M.H. (2012). Does living by the coast improve health and wellbeing?. Health Place.

[29] DfT (2019). National Travel Survey. https://www.gov.uk/government/statistics/national-travel-survey-2017.

[30] DfT (2018). TAG Data Book. https://www.gov.uk/government/publications/tag-data-book.

[31] Ward F.A., Loomis J.B. (1986). The Travel Cost Demand Model as an Environmental Policy Assessment Tool: A Review of Literature. West. J. Agric. Econ..

[32] Office for National Statistics Population Estimates. https://www.ons.gov.uk/peoplepopulationandcommunity/populationandmigration/populationestimates.

[33] Keniger L.E., Gaston K.J., Irvine K.N., Fuller R.A. (2013). What are the benefits of interacting with nature?. Int. J. Environ. Res. Public Health.

[34] Brymer E., Davids K., Mallabon L. (2014). Understanding the psychological health and well-being benefits of physical activity in nature: An ecological dynamics analysis. Ecopsychology.

[35] Gladwell V.F., Brown D.K., Wood C., Sandercock G.R., Barton J.L. (2013). The great outdoors: How a green exercise environment can benefit all. Extrem. Physiol. Med..

[36] Schiessel Harvey N. (2019). The Value of Inland Waterways.

[37] CURE Project Team (2011). Accessible Natural Green Space Standards in Towns and Cities: A Review and Toolkit for their Implementation.

[38] Saunders L.E., Green J.M., Petticrew M.P., Steinbach R., Roberts H. (2013). What are the health benefits of active travel? A systematic review of trials and cohort studies. PLoS ONE.

[39] Pang B., Kubacki K., Rundle-Thiele S. (2017). Promoting active travel to school: A systematic review (2010–2016). BMC Public Health.

[40] Rigolon A., Browning M.H.E.M., McAnirlin O., Yoon H. (2021). Green Space and Health Equity: A Systematic Review on the Potential of Green Space to Reduce Health Disparities. Int. J. Environ. Res. Public Health.

[41] Caputo A. (2017). Social desirability bias in self-reported well-being measures: Evidence from an online survey. Univ. Psychol..

[42] White M.P., Elliott L.R., Grellier J., Economou T., Bell S., Bratman G.N., Cirach M., Gascon M., Lima M.L., Lõhmus M. (2021). Associations between green/blue spaces and mental health across 18 countries. Sci. Rep..

[43] Elliott L.R., White M. (2020). BlueHealth International Survey Methodology and Technical Report.

